# Supplementary material for: Fear of missing out, social media influencers, and the social, psychological and financial wellbeing of young consumers
Source: PLoS One. 2025 Apr 15;20(4):e0319034. doi: 10.1371/journal.pone.0319034 (PMC11999132; doi:10.1371/journal.pone.0319034)
Supplement: S1 Appendix — (DOCX) [file pone.0319034.s001.docx]

**Supporting information files**

**Fear of Missing out, Social Media Influencers, and the Social, Psychological and Financial Wellbeing of Young Consumers**

# S1 Appendix. Survey Questionnaire.

(DOCX)

# S1 Appendix. Survey questionnaire.

Title: “Survey about how social media influences your shopping behavior.”

**Screener Questions**

*Questions about your social media usage frequency*.

We define social media as a platform that facilitates the sharing of ideas, thoughts, and information through virtual networks and communities which allow you to follow specific individuals, companies, or products. These include, but are not limited to: Facebook, Instagram, Twitter, Snapchat, Influenster, TikTok, Pinterest, and YouTube, etc. We do not include messaging services, like Messenger, GroupMe, or WhatsApp, or professional services, like LinkedIn. On average, how frequently do you open a social media application (Facebook, Instagram, Twitter, TikTok, Snapchat, etc.)?

Based on the definition above, on average, how frequently do you access social media?

- Hourly
- Daily
- Weekly
- Monthly
- I do not have social media (Logic to: Exit survey)

*Question about the users you follow on social media.*

A social media influencer is a person who has gained fame by successfully branding themselves as experts on social media platforms. They enthusiastically share self-generated content on topics like beauty, fitness, food, and fashion and are not famous by other means, as through sports or entertainment (they are not professional athletes or movie stars).

How many influencer accounts do you follow? Please give your best guess.

- I do not follow influencers. (Logic to: Exit survey)
- 1-5
- 6-10
- 10-15
- 16-20
- 21 or more

Are you between the ages of 18 and 40?

- Yes
- No (Logic to: Exit survey)
- Prefer not to respond (Logic to: Exit survey)

Do you presently live in the United States?

- Yes
- No (Logic to: Exit survey)
- Prefer not to respond (Logic to: Exit survey)

**Main Questionnaire: Social Media Questions**

*Questions about your social media usage.*

As a reminder, we define social media as a platform that facilitates the sharing of ideas, thoughts, and information through virtual networks and communities which allow you to follow specific individuals, companies, or products. These include, but are not limited to: Facebook, Instagram, Twitter, Snapchat, Influenster, TikTok, Pinterest, and YouTube, etc. We do not include messaging services, like Messenger, GroupMe, or WhatsApp, or professional services, like LinkedIn.

On average, how frequently do you post content on your own social media accounts?

- Everytime that I log on
- At least once a day
- At least once a week
- At least once a month
- At least once a year
- Never

On average, how frequently do you engage with (liking, commenting, etc.) posts that you see while on asocial media application?

- Everytime that I log on
- At least once a day
- At least once a week
- At least once a month
- At least once a year
- Never

I follow influencers to stay up-to-date on the following categories: (Select all that apply.)

- Beauty trends and products, including haircare, skincare, and makeup
- Health trends and products, including vitamins and diet tips
- Food trends and products, including recipes and kitchen tools
- Hospitality trends and products, including vacation and restaurant destinations
- Fashion trends and products, including retailers to shop from
- Sales on products as some influencers are offered a special discount code or make me aware of sales

*FoMO*

Questions about your personal experience with friendships.

Scale: 1 – Not at all True of Me to 5 – Extremely True of Me

- - I fear others have more rewarding experiences than me.
  - I fear my friends have more rewarding experiences than me.
  - I get worried when I find out my friends are having fun without me.
  - I get anxious when I don’t know what my friends are up to.
  - It is important that I understand my friends ‘‘in jokes’’.
  - Sometimes, I wonder if I spend too much time keeping up with what is going on.
  - It bothers me when I miss an opportunity to meet up with friends.
  - When I have a good time it is important for me to share the details online (e.g. updating status).
  - When I miss out on a planned get-together it bothers me.
  - When I go on vacation, I continue to keep tabs on what my friends are doing.

*PSIs*

Please think about a social media influencer you follow when answering these questions. If you follow multiple influencers, please think about your favorite influencer.

Scale: 1 – Strong Disagree to 5 – Strongly Agree

- - The influencer I follow make me feel as if I am with a someone I know well.
  - If the influencer I follow appeared on a TV program, I would watch that program.
  - I see the influencer I follow as natural down -to-earth people.
  - I would like to meet the influencer I follow in person.
  - I feel that I understand the emotions of the influencer I follow and share in their experiences.
  - I find myself thinking about the influencer I follow on a regular basis.
  - I like to see the influencer I follow post on social media.
  - I have been seeking out information in the media to learn more about the influencer.
  - The influencer I follow understand the kinds of things I want to know and read about.
  - I sometimes comment on the posts of the influencer that I follow.
  - I am very much aware of the details of the influencer’s life.
  - I look forward to seeing the influencers I follow post on social media.

*Social Shopping*

Thinking about the same social media influencer from the previous questions,

please indicate how much you agree or disagree on the following statements, using the following scale: (1) strongly disagree to (5) strongly agree.

- I will consider the shopping experiences of social media influencers when I want to shop.
- I will seek advice from social media influencers before I go shopping.
- I am willing to buy products recommended by social media influencers.
- I would consider buying items that the social media influencer recommends.
- I am willing to buy products recommended by the social media influencer.
- I will seek advice from the social media influencer because I go shopping.

*Questions about your shopping habits.*

Please indicate how often your shopping habits are described by the statements below, using the following scale: (1) strongly disagree to (5) strongly agree.

- - When I go shopping, I buy things that I had not intended to purchase.
  - I avoid buying things that are not on my shopping list.
  - When I see something that really interests me, I buy it without considering the consequences.
  - It is fun to buy spontaneously.
  - I am a person who makes unplanned purchases.

*Psychological Wellbeing*

Questions about your personal feelings and beliefs.

Please indicate how often you experience the following feelings or emotions, using the following scale: (1) never to (5) always.

- - I feel in control of my life.
  - I feel happy with myself as a person.
  - I feel depressed or anxious.
  - I feel I have a purpose in life.
  - I feel I am able to enjoy life.
  - I feel able to grow and develop as a person.
  - I am happy with myself and my achievements.
  - I feel I am able to live my life the way I want.
  - I feel confident in my own opinions and beliefs.
  - I am happy with my looks and appearance.
  - I feel able to do the things I choose to do.

*Social Wellbeing*

Questions about your social experiences.

Please indicate how well the following statements describe you using the following scale: (1) strongly disagree to (5) strongly agree.

- - It is easy for me to relate to others.
  - I have someone to share my feelings with.
  - I feel isolated from other people.
  - I have a strong need to belong.
  - I find it easy to get in touch with others when I need to.
  - I feel like I just don’t belong.
  - I feel I spend enough time involved in social activities.
  - I feel alone and friendless.
  - When with other people, I felt separate from them.
  - Overall, I feel that my relationships are fulfilling.

*Financial Wellbeing*

Questions about your personal finances.

How well does this statement describe you or your situation?

- - Because of my money situation, I feel like I will never have the things I want in life.
  - I am concerned that the money I have or will save won’t last.
  - I am just getting by financially.
  - I can enjoy life because of the way I’m managing my money.
  - I could handle a major unexpected expense.

How often does this statement apply to you?

- - Giving a gift for a wedding, birthday or other occasion would put a strain on my finances for the month.
  - I am behind with my finances.
  - My finances control my life.
  - I have money left over at the end of the month.

*Demographics*

Please select your age.

[Scroll age chooses from 18 to 40.]

Please select your gender.

- - Male
  - Female
  - Transgender
  - Non-binary/Non-conforming
  - Prefer not to respond

What is your marital status?

- - Never Married
  - Married
  - Separated
  - Divorced
  - Widowed
  - Prefer not to respond

Which of the following best describes you?

- - Asian or Pacific Islander
  - African American/Black
  - Caucasian/White
  - Hispanic or Latino/a/x
  - Native American, Indigenous or Aboriginal People
  - Multiracial or Biracial
  - Other race/ethnicity not listed
  - Prefer not to respond

What is your highest level of education?

- - Less than a high school diploma
  - High school diploma/GED/Alternative school completion
  - Some college credit, no degree
  - Associates degree (for example: AA, AS)
  - Bachelor’s degree (for example: BA. BS)
  - Master’s degree (for example: MA, MS, MEng, MEd, MSW, MBA)
  - Professional degree beyond bachelor’s degree (for example: MD, DDS, DVM, LLB, JD)
  - Doctorate degree (for example, PhD, EdD)
  - Prefer not to respond

What is your current employment status?

- - Employed, full-time
  - Employed, part-time
  - Employed, but temporarily furloughed
  - Unemployed, looking for work
  - Unemployed, not looking for work
  - Disabled, unable to work
  - Retired
  - Homemaker
  - Full-time caregiver for family member
  - Prefer not to respond

How many people, including yourself, live in your house?

- - 1
  - 2
  - 3
  - 4
  - 5 or more
  - Prefer not to respond

Please enter your estimated household pre-tax 2021 income.

- - Less than $25,000
  - $25,000 - $49,999
  - $50,000 - $74,999
  - $75,000 - $99,999
  - $100,000 - $149,999
  - $150,000 - $199,999
  - $200,000 and above
  - Prefer not to respond
