# Supplementary material for: Fear of missing out, social media influencers, and the social, psychological and financial wellbeing of young consumers
Source: PLoS One. 2025 Apr 15;20(4):e0319034. doi: 10.1371/journal.pone.0319034 (PMC11999132; doi:10.1371/journal.pone.0319034)
Supplement: Attachment — Submitted filename: PONE-D-24-22539 Response to Reviewers 2025.01.23.docx [file pone.0319034.s003.docx]

PONE-D-24-22539

Fear of missing out, social media influencers, and the social, psychological, and financial well-being of young consumers

PLOS ONE

Dear Dr. Sudzina and reviewers,

We appreciate the opportunity to revise our manuscript and would like to thank you and the reviewers for your valuable feedback. Below, we provide detailed responses to each comment raised by the reviewers and outline the revisions made to the manuscript accordingly. All changes have been highlighted in the revised manuscript for your convenience.

**Editor’s comments:**

1. PLOS ONE's style requirements:
   - We formatted the manuscript and title page to follow PLOS ONE’s style guidelines.
2. Financial Disclosure Statement:
   - We would like to revise the financial disclosure statement to: "The authors acknowledge generous funding from the Coca-Cola Critical Difference for Women Grant for Research on Women, Gender, and Gender Equity from The Women’s Place at The Ohio State University. The funders had no role in study design, data collection and analysis, decision to publish, or preparation of the manuscript."
3. Acknowledgments Section:
   - We have removed funding-related text from the Acknowledgments section.
4. Data Availability Statement:
   - The entire data are freely accessible from the OSF.IO database (URL: <https://osf.io/v5zw6/?view_only=3fb68ef318e14d069aa2531de96652f0> ).
1. References:
   - We have reviewed the reference list for completeness and accuracy and ensured that no retracted articles are cited. Any updates to the references are reflected in the manuscript.

**Reviewer #1 Comments:**

1. Section 2: "Materials and Methods" should be named "Methods," with two sub-sections: "Participants and Procedure" and "Measures."
   - We have renamed Section 2 to "Methods" and added two sub-sections including “Participants and Procedure” and “Measures.”
2. In Section 3: "Results," authors should add a sub-section "Descriptive Statistics and Correlations," with data summarized in two tables.
   - We have added a sub-section titled "Descriptive Statistics" under the "Results" section and Table 1 summarizing the descriptive statistics.
   - Table 3 under the measurement model section presents the correlation matrix.
3. Sub-section 5.2: "Limitations and Suggestions for Future Studies" should be moved to the end of Section 4: "Discussion," as sub-section 4.1.
   - The "Limitations and Suggestions for Future Studies" section has been relocated to the end of the discussion section.

**Reviewer #2 Comments:**

1. Figure 1 is hard to read with arrows stacked on top of each other for the three well-being boxes. Try to move them apart.
   - We have revised Figs 1 and 2 to provide more space between elements, ensuring better readability.
2. "Mturk workers who lived in the United States were eligible to participate in..." - were or were not?
   - We confirm that the original statement is correct: "Mturk workers who lived in the United States were eligible to participate in the survey."
3. "In particular, we limited the sample to those aged 18 to 40 because these ages represent Gen Z and Millennials who use social media more frequently than other generations." - Millennials age range extends beyond 40 in 2022
   - We acknowledge that the Millennials' age range extends slightly beyond 40 in 2022. To address this, we have revised the sentence to: “In particular, we limited to sample to individuals aged 18 to 40, as this group represents young adult consumers who tend to use social media more frequently than older age group.”
4. Considering your sample had relatively low mode income range and score low on the self-reported financial well being measure, these demographic variables might explain why social shopping has no significant impact on any of their well being measures. Consider adding brief explanation of this in conclusion
   - We added the following statement in the discussion section: “Alternatively, the relatively low modal income range and low self-reported financial well-being scores in the current sample may explain why social shopping had no significant impact on their well-being measures.”

We trust that these revisions address the concerns raised by the reviewers and enhance the quality of our manuscript. Thank you again for your constructive feedback and for considering our work for publication in PLOS ONE. We look forward to your response.

Sincerely,

Cäzilia Loibl
